# Touch imprint cytology with massively parallel sequencing (TIC‐seq): a simple and rapid method to snapshot genetic alterations in tumors

**DOI:** 10.1002/cam4.950

**Published:** 2016-10-24

**Authors:** Kenji Amemiya, Yosuke Hirotsu, Taichiro Goto, Hiroshi Nakagomi, Hitoshi Mochizuki, Toshio Oyama, Masao Omata

**Affiliations:** ^1^ Genome Analysis CenterYamanashi Prefectural Central Hospital1‐1‐1 FujimiKofuYamanashi400‐8506Japan; ^2^ Pathology DivisionLaboratory DepartmentYamanashi Prefectural Central Hospital1‐1‐1 FujimiKofuYamanashi400‐8506Japan; ^3^ Lung Cancer and Respiratory Disease CenterYamanashi Prefectural Central Hospital1‐1‐1 FujimiKofuYamanashi400‐8506Japan; ^4^ Department of Breast SurgeryYamanashi Prefectural Central Hospital1‐1‐1 FujimiKofuYamanashi400‐8506Japan; ^5^ The University of Tokyo7‐3‐1 Hongo, Bunkyo‐kuTokyo113‐8655Japan

**Keywords:** Cytology, FFPE, heterogeneity, mutation, next‐generation sequencing, tumor

## Abstract

Identifying genetic alterations in tumors is critical for molecular targeting of therapy. In the clinical setting, formalin‐fixed paraffin‐embedded (FFPE) tissue is usually employed for genetic analysis. However, DNA extracted from FFPE tissue is often not suitable for analysis because of its low levels and poor quality. Additionally, FFPE sample preparation is time‐consuming. To provide early treatment for cancer patients, a more rapid and robust method is required for precision medicine. We present a simple method for genetic analysis, called touch imprint cytology combined with massively paralleled sequencing (touch imprint cytology [TIC]‐seq), to detect somatic mutations in tumors. We prepared FFPE tissues and TIC specimens from tumors in nine lung cancer patients and one patient with breast cancer. We found that the quality and quantity of TIC DNA was higher than that of FFPE DNA, which requires microdissection to enrich DNA from target tissues. Targeted sequencing using a next‐generation sequencer obtained sufficient sequence data using TIC DNA. Most (92%) somatic mutations in lung primary tumors were found to be consistent between TIC and FFPE DNA. We also applied TIC DNA to primary and metastatic tumor tissues to analyze tumor heterogeneity in a breast cancer patient, and showed that common and distinct mutations among primary and metastatic sites could be classified into two distinct histological subtypes. TIC‐seq is an alternative and feasible method to analyze genomic alterations in tumors by simply touching the cut surface of specimens to slides.

## Introduction

In the clinical setting, formalin‐fixed paraffin‐embedded (FFPE) tumor specimens are commonly used for pathological diagnosis. Together with the development of molecular targeting drugs, the genetic analysis of *HER2* amplification and somatic mutations in *KRAS* and *EGFR* is important for so‐called “precision medicine” and to help make therapeutic decisions 1, 2, 3. Recent advances in sequencing technology have led to the identification of genetic alterations in several tumor types using FFPE specimens. However, formalin fixation‐induced DNA‐protein cross‐linking and DNA fragmentation hinders the ability to detect all genetic aberrations 4, 5, 6. Additionally, DNA derived from FFPE tissue is often extensively fragmented during preparation by approaches such as xylene treatment, paraffin embedding, and heat incubation; so, such DNA samples may not be suitable for genetic analysis. Furthermore, it takes several days to prepare these DNA samples from FFPE tissue; therefore, it is necessary to improve the technical method for rapidly obtaining accurate genetic results to aid bench–to‐bedside decisions.

Cytology is an alternative method of pathological diagnosis involving simple specimen preparation without the need for serial sections from FFPE samples. Instead of formalin fixation, and the use of ethanol and Papanicolaou (Pap) staining, cytology uses methanol followed by air‐drying with May‐Grünwald–Giemsa (Giemsa) staining. Because of the differences in fixation reagents, larger amounts of intact and high‐quality DNA can be obtained from cytology samples. The touch imprint cytology (TIC) technique was developed in the 1940s and can be used for diagnosis 7. TIC is routinely performed on sentinel lymph nodes and marginal tissues from breast cancer patients for intraoperative rapid diagnosis 8, 9. Moreover, on‐site TIC was recently shown to be a useful tool for the evaluation of sample adequacy and preliminary diagnosis in various types of cancer 10, 11, 12. Cytology‐derived DNA is also used for next‐generation sequencing analysis 13, 14, 15, 16, 17, 18; however, few reports have used paired TIC and corresponding tumor tissue to comprehensively detect somatic mutations and to compare mutational profiles between TIC and tumor tissues.

This study examined the utility of cytology‐derived DNA in the detection of somatic mutations by targeted sequencing with a multigene panel. We also assessed an alternative method, touch imprint cytology with massively parallel sequencing (TIC‐seq), to determine whether it faithfully detects somatic mutations in tumors.

## Materials and Methods

### Patients and sample preparation

TIC and FFPE samples were obtained between December 2014 and November 2015 from nine patients with lung cancer (cases 1–9: six adenocarcinomas, three squamous cell carcinomas) and one breast cancer patient (case 10). Mutations in the epidermal growth factor receptor gene (*EGFR*) were examined by the PCR‐Invader assay with FFPE DNA prior to our analysis in the six lung adenocarcinoma cases. Buffy coats were isolated after centrifugation at 820*g* at 25°C for 10 min and stored at −80°C until DNA extraction. Buffy coat DNA was extracted with the QIAamp DNA Blood Mini QIAcube kit using a QIAcube instrument (Qiagen, Hilden, Germany), and the concentration was determined with a NanoDrop 2000 spectrophotometer (Thermo Fisher Scientific, Waltham, MA). This study was approved by the institutional review board at our hospital. Written informed consent was obtained from all patients who participated in this study.

### TIC preparation

TIC samples were made by touching the cut surface of 5 mm^2^ to 1 cm^2^ fresh tumor tissue from surgically resected lung and primary breast carcinomas to slide glass. Metastatic breast carcinomas were obtained from cut lymph node surfaces. Over 80% of the slide surface was touched for each tissue using the Arcturus PEN Membrane Glass Slide (Thermo Fisher Scientific) for laser capture microdissection. Lung carcinoma samples were prepared on air‐dried slides for Giemsa staining and fixed with 95% ethanol on slides for Pap staining, while breast carcinoma slides were prepared on air‐dried slides only for Giemsa staining.

### Cytological staining

To assess tumor cellularity, all samples were stained with Cyto Quick A solution (Muto Pure Chemicals, Tokyo, Japan) for 5 sec, then stained with Cyto Quick B solution for 15 sec. TIC samples were stained with Pap or Giemsa stain. Pap staining was performed by placing samples in the following solutions in order: 70% ethanol for 30 sec, water for 50 sec, hematoxylin for 2 min, water for 1 min, 70% ethanol for 30 sec, 0.3% hydrochloric acid/70% ethanol for 15 sec, water for 3 min, 70% ethanol for 30 sec, 95% ethanol for 30 sec ×2, OG6 solution (Muto Pure Chemicals) for 2 min, 95% ethanol for 30 sec ×3, EA50 solution (Muto Pure Chemicals) for 3 min, 95% ethanol for 30 sec ×2, 100% ethanol for 30 sec ×3, and xylene for 30 sec ×3. Giemsa staining was performed using the following solutions: May‐Grünwald solution (Muto Pure Chemicals) for 3 min, water for 1 min, Giemsa solution (Muto Pure Chemicals) for 15 min, and water for 1 min. Slides were stored at 4°C until required for DNA extraction.

### FFPE tissue preparation

Serial sections (10 *μ*m thick) were prepared from FFPE samples and stored at room temperature overnight. The sections were then deparaffinized and stained with hematoxylin‐eosin (HE) as follows: xylene for 5 min ×2, 100% ethanol for 30 sec, 95% ethanol for 30 sec, 70% ethanol for 30 sec, water for 30 sec, Lillie–Mayer hematoxylin (Muto Pure Chemicals) for 30 sec, water for 30 sec, eosin Y (Merck, Tokyo, Japan) for one dip, 70% ethanol for 30 sec, 95% ethanol for 30 sec, 100% ethanol for 30 sec, and xylene for 5 min ×2. All slides were reviewed by a pathologist and cytotechnologist to check sample adequacy and tumor cellularity. These slides were also stored at 4°C until DNA extraction.

### Laser capture microdissection

Laser capture microdissection was performed using an Arcturus XT laser microdissection system (Thermo Fisher Scientific) to enrich tumor cellularity. When the average percentage of tumor cells was more than 70%, cells were collected by resecting the entire slide with a blade. DNA extraction from TIC and FFPE samples was performed with a QIAamp DNA FFPE kit (Qiagen) according to the manufacturer's instructions.

### Immunohistochemical analysis

Immunohistochemical analysis was performed on breast carcinoma specimens. Sections were deparaffinized, and antigen activation was performed by heat treatment in EDTA solution at pH 8.0. Protein expression was evaluated on 3‐*μ*m‐thick FFPE sections with anti‐estrogen receptor (ER) (SP1; Ventana, Tucson, AZ), anti‐progesterone receptor (PgR) (1E2; Ventana), and antihuman epidermal growth factor receptor 2 (HER2, also known as ERBB2) (4B5; Ventana) antibodies using the Ventana BenchMark XT system (Roche, Tucson, AZ). All sections were evaluated by a pathologist.

### DNA quality analysis

The integrity of purified DNA from TIC and FFPE samples was assessed using the TaqMan RNase P Detection Reagents Kit and the FFPE DNA QC Assay v2 on a ViiA 7 Real‐Time PCR System (Thermo Fisher Scientific). DNA quality analysis was performed as described previously 19. Briefly, the PCR reaction mix was transferred into a MicroAmp Fast Optical 96‐Well Reaction Plate. Two primer pairs were used to amplify a short‐ (87 bp) and a long‐ (268 bp) amplicon. Human control genomic DNA (included with the TaqMan RNase P Detection Reagents Kit) was serially diluted four times for a five‐point standard curve and the absolute DNA concentrations were determined. The following PCR protocol was used: 95°C for 20 sec, followed by 45 cycles of 95°C for 1 sec and 60°C for 20 sec. Assessment of DNA fragmentation was estimated with the ratio of DNA (relative quantification; RQ) obtained for the long amplicon to the short amplicon. The RQ value was an indicator of the degradation level of genomic DNA; a higher RQ value indicates better quality genomic DNA.

### Targeted sequencing

Template DNA was extracted from cytology specimens, which were stained with Pap and Giemsa for lung cancer cases, and with Giemsa for the breast cancer case. Targeted sequencing was performed as described previously 20, 21. In brief, multiplex PCR was performed using the Ion AmpliSeq Library Kit 2.0 with custom panels designed by Ion AmpliSeq designer software (Thermo Fisher Scientific). The lung cancer panel targets 53 significantly mutated genes (2896 primer pairs) 19, while the breast cancer panel targets 53 genes (2863 primer pairs). Multiplex PCR products were partially digested with FuPa reagent and subsequently ligated with barcodes using an Ion Xpress Barcode Adapters kit (Thermo Fisher Scientific). The ligated library was purified with Agencourt AMPure XP reagents (Beckman Coulter, Brea, CA), and the library concentration was determined using an Ion Library Quantitation Kit (Thermo Fisher Scientific), then each library was diluted to 10 pmol/L and the same amount of library was pooled for one sequence reaction. Emulsion PCR was carried out using the Ion OneTouch System and Ion PGM Template OT2 200 Kit or Ion PI Template OT2 200 Kit v3 (Thermo Fisher Scientific). Template‐positive Ion Sphere Particles were then enriched using an Ion OneTouch ES system (Thermo Fisher Scientific). Purified Ion Sphere particles were loaded onto an Ion 318 Chip v2 or PI Chip (Thermo Fisher Scientific) and massively parallel sequencing was carried out on an Ion PGM System or Ion Proton (Thermo Fisher Scientific).

### Data analysis

Sequence data were processed using standard Ion Torrent Suite Software running on the Torrent Server, as previously described 22. Raw signal data were analyzed using Torrent Suite version 4.0. The pipeline included signaling processing, base calling, quality score assignment, adapter trimming, PCR duplicate removal, read alignment to human genome 19 reference (hg19), quality control of mapping quality, and coverage analysis. Nonsynonymous somatic mutations and splice site mutations were identified by the Ion Reporter Server System (Thermo Fisher Scientific), and peripheral blood DNA was used as a control to detect variants in tumors (Tumor–Normal pairs). Mutations were filtered according to the parameters of the minimum count for mutant allele reads ≥5, coverage depth ≥10, variant allele faction ≥10%, UCSC Common SNPs = Not In, and Confident Somatic Variants = In. High confident somatic mutations were defined as mutations harboring a variant allelic fraction of over 20% in either TIC or FFPE samples. Copy number data were obtained from the Tumor–Normal pair algorithm and filtering using the parameters of confidence value ≥20 and precision value ≥20. If somatic mutations were called, sequence data were visually confirmed with the Integrative Genomics Viewer (IGV) and any sequence, alignment, or variant call error artifacts were discarded.

### Cellular prevalence analysis

Cellular prevalence was inferred using the PyClone algorithm (version 0.12.3) 23. The allelic fraction of somatic mutations was obtained using the number of reference reads and the number of variant reads. Copy number data of each mutation position were used in this analysis. For multiple sample analysis, only somatic mutations were shared among tumor samples. PyClone was run with 10,000 iterations and a burn‐in of 1000, as suggested by the authors (http://compbio.bccrc.ca/software/pyclone/).

### Phylogenetic tree

The rooted phylogenetic tree was constructed using the Unweighted Pair Group Method with Arithmetic Mean (UPGMA) based on the Euclidean distance. The analysis of phylogenetics and evolution (APE) package for R was used to show the evolutionary findings.

### Statistical analysis

The Bonferroni correction was used to calculate the adjusted *P*‐value for multiple pairwise comparisons using the R statistical package (version 3.1.2) (http://www.r-project.org/).

## Results

### DNA prepared using the touch imprint technique is intact

**Figure 1:**
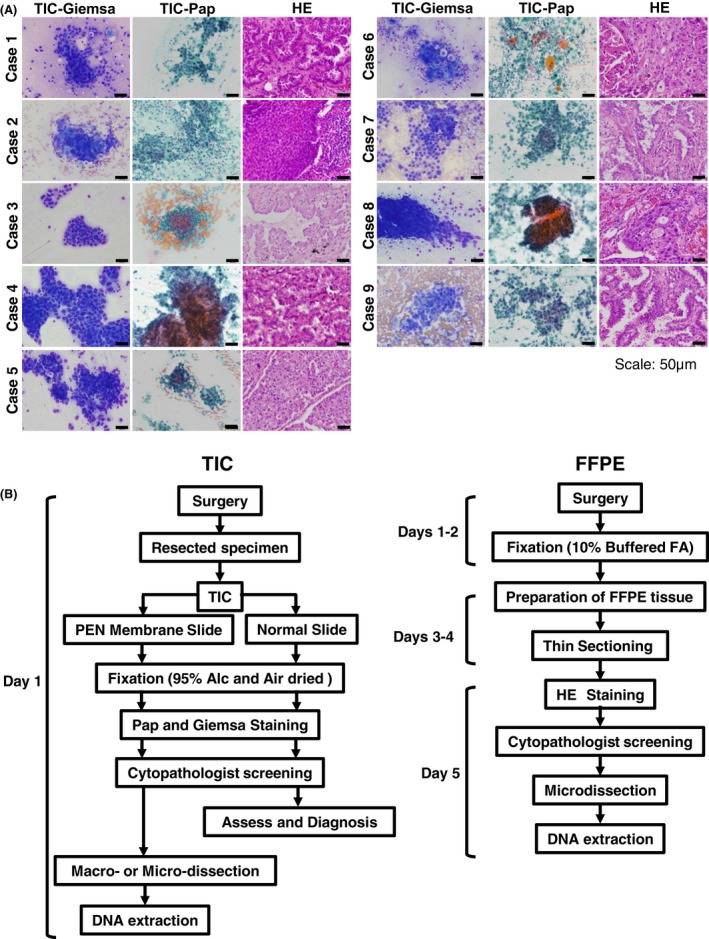
Preparation of touch imprint cytology (TIC) and formalin‐fixed paraffin‐embedded (FFPE) samples for targeted sequencing. (A) Representative images of TIC and HE staining from lung cancer samples (*n* = 9). TIC samples were stained with Giemsa or Pap staining reagent. TIC samples showed similar morphology in corresponding histopathological specimens. (B) Flow chart of TIC and FFPE sample preparation. DNA extraction takes at least 4 days from FFPE tissues, but only 1 day from TIC. Alc, alcohol; FA, formalin; HE, hematoxylin‐eosin.

We prepared 18 TIC (nine Giemsa; nine Pap) and nine FFPE samples from lung cancer patients (*n* = 9) (Fig. 1A). The scheme for preparing TIC and FFPE samples is described in Figure 1B. In the standard protocol, it takes at least 4–7 days to obtain DNA from FFPE specimens during resected tissue fixation and processing. In contrast, TIC was completed within 1 day and discriminated tumor cells from normal cells by staining (Fig. 1B). The average number of slides required for DNA extraction was one for TIC and 3.4 for FFPE (Table S1). Thus, TIC is a simpler and more rapid method for sample preparation compared with the use of FFPE samples.

**Figure 2:**
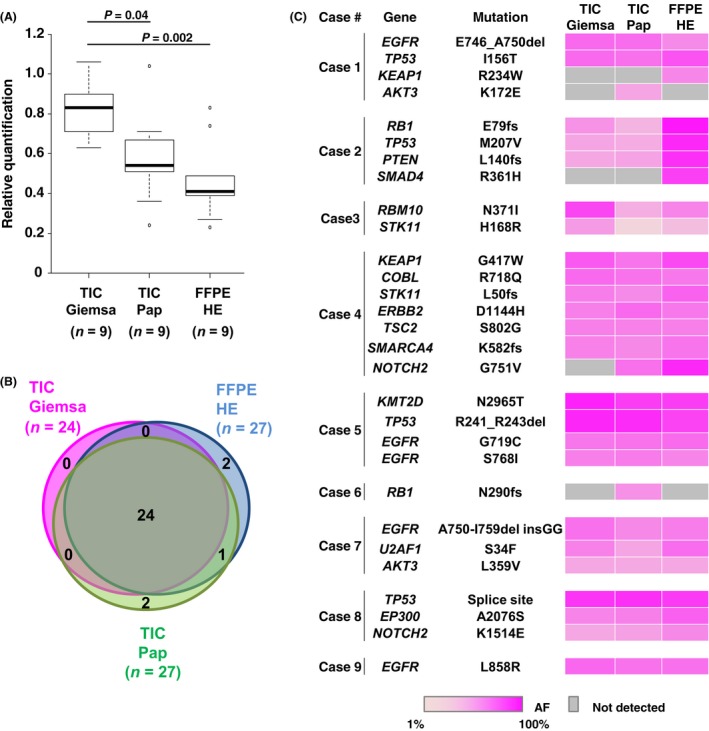
Touch imprint cytology (TIC)‐seq accurately detected somatic mutational profiles in lung cancers. (A) Comparison of relative quantification values among three sample preparation approaches: TIC‐Giemsa, TIC‐Pap, and formalin‐fixed paraffin‐embedded (FFPE)‐hematoxylin‐eosin (HE) samples. In the box plots, the bottom and top of each box correspond to the first and third quartiles, respectively, and the line inside is the median. *P‐*values were calculated by the Bonferroni method. (B) Venn diagrams of three sample preparation types revealed that most mutations overlapped between TIC‐Giemsa (pink), TIC‐Pap (green), and FFPE‐HE samples (blue). (C) Heat map showing the distribution of 78 somatic mutations for each sample preparation method from nine lung cancer patients. Values of allelic fractions are indicated in the graduated color scale from 1% (light pink) to 100% (pink). Gray columns showed no identified mutation.

To examine whether TIC DNA is applicable for genomic analysis, we assessed the DNA quality and quantity by quantitative real‐time PCR. The amount of long DNA (268 bp) in TIC samples was greater than that of FFPE DNA per slide ((TIC with Giemsa staining (TIC‐Giemsa), average: 1009.3 ng (range, 3.6–3947.3 ng); TIC with Pap staining (TIC‐Pap), average: 593.2 ng (range, 9.7–1428.1 ng); FFPE, average: 90.4 ng, (range, 15.7–222.0 ng)) (Table S2). The RQ value of TIC‐Giemsa DNA was higher than that of TIC‐Pap DNA or FFPE DNA (Fig. 2A), suggesting that the TIC‐Giemsa DNA was relatively unfragmented.

### TIC‐seq captures genetic alterations in tumors

To examine whether somatic mutations in tumor tissue could be detected using TIC DNA, we performed targeted sequencing of 53 significantly mutated lung cancer‐associated genes 19. After checking the DNA quality as described above, the targeted sequence library was prepared. Sequencing quality data were equivalent between FFPE DNA and TIC DNA (Table 1). The average coverage depth was 864× (range, 636–1060×) for TIC‐Giemsa DNA, 641× (range, 419–956×) for TIC‐Pap DNA, and 717× (range, 524–838×) for FFPE DNA (Table 1 and Table S3).

**Table 1:** Summary of targeted sequence quality data

	TIC‐Giemsa (*n *=* *9)	TIC‐Pap (*n *=* *9)	FFPE (*n *=* *9)
Mapped reads (millions)	2.68 ± 0.30	1.93 ± 0.42	2.23 ± 0.39
On Target (%)	97 ± 0.005	97 ± 0.4	97 ± 0.6
Mean Depth	864 ± 112	641 ± 185	717 ± 164
Mean Read Length (bp)	107 ± 4	107 ± 7	113 ± 7
≥Q20/Total Base (%)	83 ± 2	82 ± 2	82 ± 1

Values are mean ± standard deviation. FFPE, formalin‐fixed paraffin‐embedded; TIC, touch imprint cytology.

We next compared the high confident somatic mutations in TIC and FFPE DNA to estimate the specificity for detecting somatic mutations in tumors. In total, 78 highly confident mutations were identified by targeted sequencing (24 mutations in TIC‐Giemsa, 27 mutations in TIC‐Pap, and 27 mutations in FFPE). Among these, 24 somatic mutations were consistent in each sample (92% concordance, 72/78) (Fig. 2B and C). *EGFR* mutations in six lung adenocarcinomas were 100% concordant according to data from TIC‐seq and the PCR‐Invader assay (Table S4). These results suggested that TIC‐seq precisely recaptured tumor somatic mutations.

### Multiregional analysis by TIC‐seq reveals tumor heterogeneity

**Figure 3:**
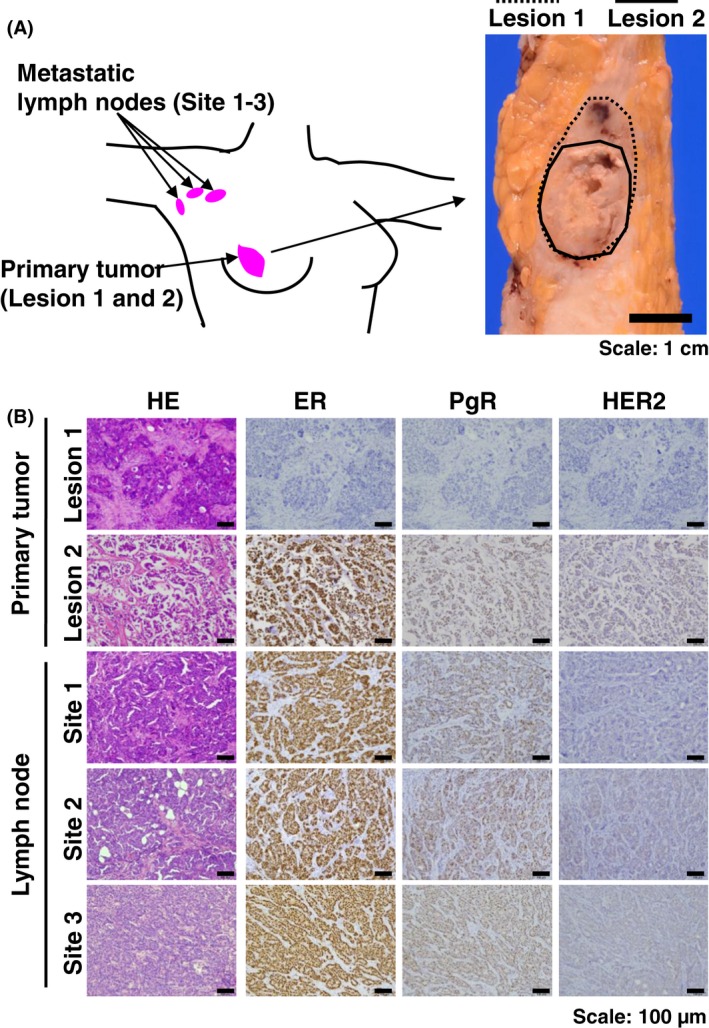
Breast cancer patient with different histological subtypes in primary and metastatic sites. (A) Pink circles indicate primary breast cancer and lymph node metastatic sites at levels I and II. Macroscopic image of corresponding primary tumor is shown on the right. In the primary tumor, the solid line indicates the hormone receptor (HR) (‐) and HER2 (‐) site in lesion 1 and the dotted line indicates the HR (+) and HER2 (‐) site in lesion 2. Scale bar: 1 cm. (B) Representative image of HE and immunohistochemical staining. Primary tumor at lesion 1 indicates the estrogen receptor (ER)^−^ PgR^−^ HER2^−^, tumor at lesion 2 indicates ER ^+^ PgR^+^ HER2^−^, and all the metastatic lymph nodes showed ER ^−^ PgR^−^ HER2^−^. Scale bar: 100 *μ*m.

Tumors acquire heterogeneity during progression; therefore, we next examined whether TIC‐seq captures this heterogeneity. We applied TIC‐seq to a breast cancer patient with a primary tumor and three metastatic lymph nodes (Fig. 3A). Immunohistochemical analysis showed that the primary tumor exhibited two different histological features including triple‐negative components at lesion 1 (ER^−^ PgR^−^ HER2^−^) and hormone receptor positivity at lesion 2 (ER^+^ PgR^+^ HER2^−^); and all three metastasis sites were ER^+^ PgR^+^ HER2^−^ (Fig. 3B).

For genetic analysis, we prepared three samples from the primary site (two FFPE and one TIC) and four samples from metastatic lymph nodes (three FFPE and one TIC) (Fig. S1). Targeted sequencing gave an average coverage depth of 1771× (range, 1475–2114×) (Table S3).

**Figure 4:**
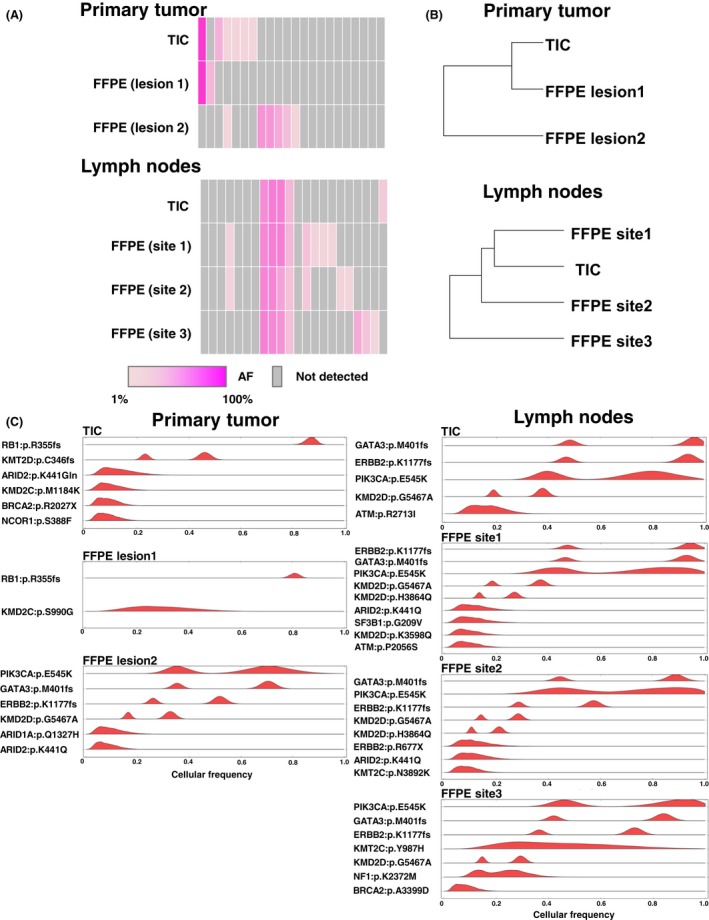
TIC‐seq discriminates clonal subpopulation in multiregional tumor site. (A) Heat map showing the distribution of 43 somatic mutations for each tumor region (*n* = 7). Values of allelic fractions are indicated in graduation color scale from 1% (light pink) to 100% (pink). Gray columns showed no identified mutation. (B) Phylogenetic tree constructed using somatic mutational profiles of the primary tumor (*n* = 3) and metastatic lymph nodes (*n* = 4). (C) Inferred cellular frequencies for seven specimens are shown as the distribution of posterior probabilities from the PyClone model (Materials and Methods). Cellular frequency distributions are shown for each somatic mutation. TIC, touch imprint cytology.

Two distinct genetic profiles were identified in the primary tumor using FFPE DNA, suggesting that the primary tumor was derived from different tumor clones, that is, intratumoral heterogeneity existed (Fig. 4A). In the TIC sample, clustering analysis showed that genetic alteration patterns were similar to one of the FFPE samples (lesion 1) but not the other (lesion 2) (Fig. 4B). Additionally, PyClone analysis inferring the cellular prevalence of each primary sample showed that the tumor clone with *RB* p.Arg355fs had a high clonality of approximately 80%–90% in FFPE at lesion 1 and in the TIC sample (Fig. 4C). These results suggested that TIC samples were mainly collected from the primary tumor for lesion 1, but not lesion 2. In the metastatic sites, the mutational profiles and the cellular prevalence of each sample were very similar among FFPE DNA (sites 1–3) and TIC (Fig. 4A and C). Clustering analysis showed that the relationship between the TIC and FFPE samples at site 1 was nearest (Fig. 4B). Interestingly, lesion 2 of the primary tumor shared similar mutational patterns with the metastatic sites, implying that tumor cells from this lesion metastasized to the lymph nodes (Fig. 4C and Fig. S2). Accordingly, these results indicate that TIC‐seq analysis is able to capture the genetic alterations of each tumor site and is a feasible assay for analyzing tumor heterogeneity.

## Discussion

In this study, we present a method for detecting somatic mutations using TIC DNA and next‐generation sequencing (TIC‐seq). TIC‐seq is a less time‐consuming and simpler method for the analysis of somatic alterations than FFPE preparation and can be applicable for examining tumor heterogeneity. Therefore, TIC‐seq is feasible for researching cancer genomics and performing molecular diagnostics in clinical settings.

We now face a new era of precision medicine that includes the development of molecular targeting drugs. With the advent of technological developments in sequencing, genetic landscapes in cancer have been revealed in several types of tumors. DNA from FFPE tissue is routinely used for genetic analyses in the clinic, and next‐generation sequencing of FFPE tissue has been successfully used to detect somatic alterations 24, 25. However, FFPE DNA is often degraded during formalin fixation. Moreover, formalin fixation can lead to artifactual single‐nucleotide changes, such as C:G>T:A 26, 27, 28. These make it difficult to discriminate true low‐frequency variants from false‐positive changes 29, 30.

In this study, we focused on the TIC technique; this is easier for specimen preparation and for the enrichment of tumor cells than FFPE tissue 31, which can take several days to extract DNA (Fig. 1B). While the tumor cells of FFPE samples were physically damaged during the preparation of serial sections, TIC‐derived tumor cells remained intact. Additionally, unlike FFPE samples, TIC uses ethanol or air‐drying for fixation, enabling high‐quality, abundant DNA to be obtained from cytological samples. Indeed, we showed that TIC DNA, especially TIC‐Giemsa, is more abundant and intact than FFPE DNA (Fig. 2A and Table S2). Because a simple method for preparing samples is important in clinical settings, TIC DNA is considered a suitable material for genetic analysis. The method is a rapid, simple, and cost‐effective tool to provide tumor‐enriched samples without expensive microdissection systems, thus enabling cancer patients to be treated earlier.

We showed that the DNA yield extracted from Giemsa‐stained samples was higher than that from Pap‐stained samples. This is likely to reflect the high cell loss from slides during fixation in ethanol in the Pap‐staining technique compared with spray fixation with isopropanol. We also observed a higher quality of TIC‐Giemsa DNA compared with TIC‐Pap DNA (Fig. 2A), as reported previously 32. Although the reasons for this are unknown, we speculate that the staining procedure affects the DNA integrity. Pap and Giemsa staining differ in their fixative and dehydration procedures: Pap staining typically uses xylene, while Giemsa staining uses air‐drying. Although we found that xylene fixation for several days leads to DNA fragmentation (data not shown), Pap staining requires specimens to be dipped in xylene for only a few minutes. Nevertheless, this short fixation with xylene may be sufficient to negatively affect DNA integrity. A more detailed comparison of the effects of staining procedures on DNA quantities and qualities is required.

We reasoned that TIC DNA combined with next‐generation sequencing would be useful for analyzing genetic alterations. We performed targeted sequencing with paired TIC and FFPE DNA from patients with lung or breast cancer, and successfully obtained sequencing data in all cases. The sequencing quality and coverage were almost comparable between TIC and FFPE DNA, and identified high confident mutations were highly concordant. This concordant result is remarkable given the procedural differences, in that the TIC‐seq technique simply touches the cut surface of fresh specimens, whereas FFPE requires microdissection of the tumor.

Previous work has conducted next‐generation sequencing analysis with only TIC samples but not corresponding FFPE specimens. Our study identified somatic mutations in paired TIC and FFPE specimens and compared these mutations in detail. We showed that the corresponding mutations were precisely detected in both FFPE and TIC (Fig. 2B), and that TIC‐seq analysis data were consistent with the results of conventional Invader‐PCR methods (Table S4). Furthermore, TIC‐seq clearly demonstrated common and distinct mutations among primary and metastatic sites. These results were supported by histopathological and immunohistochemical differences and statistical analysis between primary and metastatic tumors.

TIC‐seq has a potential application for smaller specimens including biopsies. Clinically, small specimens are usually obtained by a wide range of methods such as endoscopic biopsy, transbronchial lung biopsy, and endobronchial ultrasound‐guided transbronchial needle aspiration for tumor diagnosis. The small quantities of specimens obtained make it difficult to perform genetic analysis and derive genetic information for diagnosis. Although biopsy specimens usually contain very small amounts of tissue, TIC‐seq would still be feasible because the DNA derived from this technique is of such a good quality that next‐generation sequencing could readily be performed. Thus, TIC‐seq using endoscopic or core‐needle biopsy from advanced cancer patients is meaningful for early drug treatment and treatment selection.

## Conflict of Interest

The authors have no disclosures.

## Supporting information


**Figure S**1**.** Representative images of TIC‐Giemsa obtained from the primary tumors and metastatic lymph nodes of the breast cancer patient Scale bar: 20 *μ*m.

**Figure S**2**.** (A) Phylogenetic tree constructed using somatic mutational profiles of seven different tumor specimens. Data of somatic mutations were used as shown in Figure 4A. LN, lymph node; Primary, primary tumor. (B) Cellular prevalence of each cluster in multiple samples inferred by PyClone analysis.

**Table S1**. Number of slides prepared for DNA extraction.

**Table S2**. DNA quality data.

**Table S3**. Summary of sequencing data and coverage analysis data for each sample.

**Table S4**. Comparison of *EGFR* mutations detected by targeted sequencing and PCR‐Invader method.
