# Supplementary figures and images for: Touch imprint cytology with massively parallel sequencing (TIC‐seq): a simple and rapid method to snapshot genetic alterations in tumors
Source: Cancer Med. 2016 Oct 24;5(12):3426–36. doi: 10.1002/cam4.950 (PMC5224853; doi:10.1002/cam4.950)

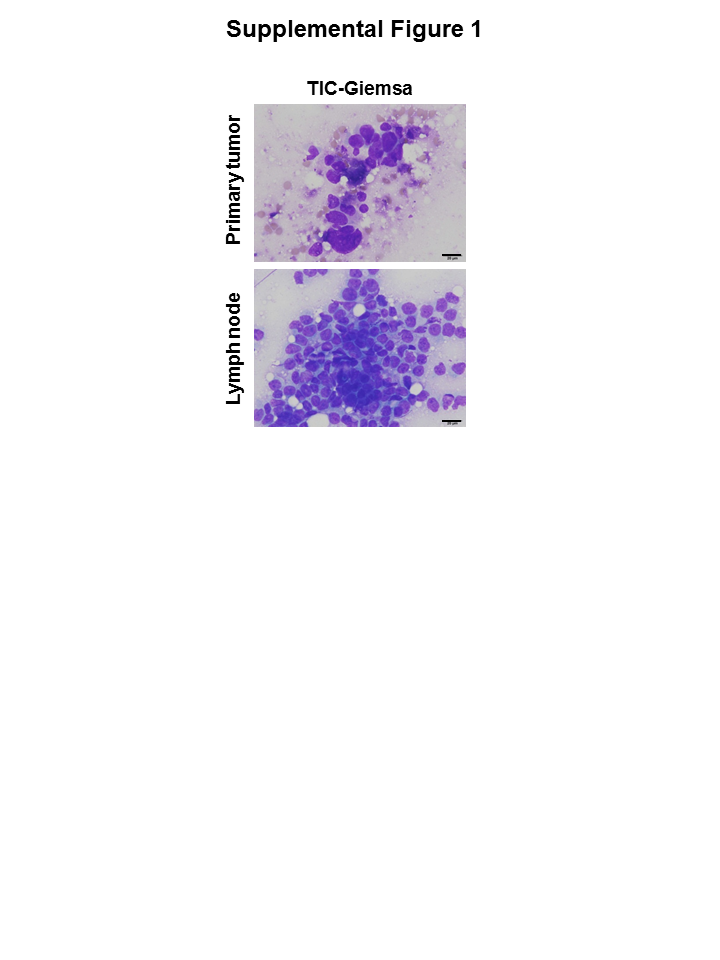

Supplement: Supplementary file 1 — Figure S1. Representative images of TIC‐Giemsa obtained from the primary tumors and metastatic lymph nodes of the breast cancer patient Scale bar: 20 μm. [file CAM4-5-3426-s001.TIF]

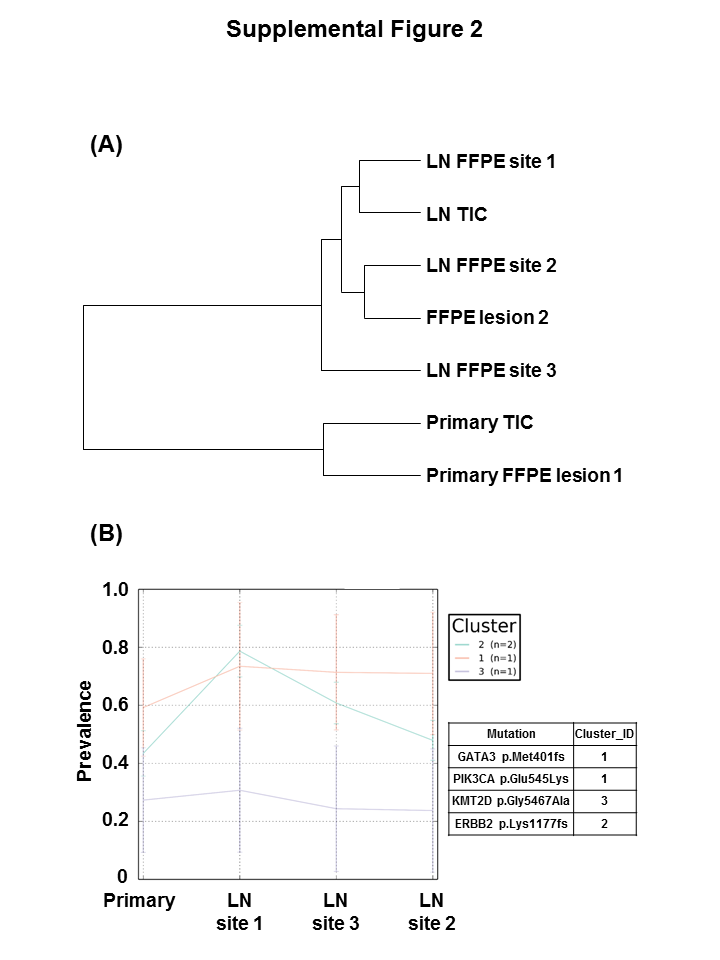

Supplement: Supplementary file 2 — Figure S2. (A) Phylogenetic tree constructed using somatic mutational profiles of seven different tumor specimens. Data of somatic mutations were used as shown in Figure 4A. LN, lymph node; Primary, primary tumor. (B) Cellular prevalence of each cluster in multiple samples inferred by PyClone analysis. [file CAM4-5-3426-s002.TIF]
