# Supplementary material for: Touch imprint cytology with massively parallel sequencing (TIC‐seq): a simple and rapid method to snapshot genetic alterations in tumors
Source: Cancer Med. 2016 Oct 24;5(12):3426–36. doi: 10.1002/cam4.950 (PMC5224853; doi:10.1002/cam4.950)
Supplement: Supplementary file 3 — Table S1. Number of slides prepared for DNA extraction. [file CAM4-5-3426-s003.docx]

**Supplemental Table 1. Number of slides prepared for DNA extraction.**

| ID | TIC | FFPE |
| --- | --- | --- |
| Case 1 | 1 | 3 |
| Case 2 | 1 | 3 |
| Case 3 | 1 | 7 |
| Case 4 | 1 | 3 |
| Case 5 | 1 | 2 |
| Case 6 | 1 | 2 |
| Case 7 | 1 | 4 |
| Case 8 | 1 | 3 |
| Case 9 | 1 | 4 |
