# Supplementary material for: Touch imprint cytology with massively parallel sequencing (TIC‐seq): a simple and rapid method to snapshot genetic alterations in tumors
Source: Cancer Med. 2016 Oct 24;5(12):3426–36. doi: 10.1002/cam4.950 (PMC5224853; doi:10.1002/cam4.950)
Supplement: Supplementary file 4 — Table S2. DNA quality data. [file CAM4-5-3426-s004.docx]

**Supplemental Table 2. DNA quality data.**

|  | TIC-Giemsa (*n*=9) | | |  | TIC-Pap (*n*=9) | | |  | FFPE-HE (*n*=9) | | | |
| --- | --- | --- | --- | --- | --- | --- | --- | --- | --- | --- | --- | --- |
|  | Total DNA  per slide (ng) | | RQ |  | Total DNA  per slide (ng) | | RQ |  | Total DNA  per slide (ng) | | | RQ |
| ID | Short | Long |  |  | Short | Long |  |  | | Short | Long |  |
| Case 1 | 462.4 | 418.5 | 0.90 |  | 665.9 | 358.6 | 0.54 |  | 180 | | 134.0 | 0.74 |
| Case 2 | 24.9 | 18.1 | 0.73 |  | 19.0 | 9.7 | 0.51 |  | 294.3 | | 222.0 | 0.83 |
| Case 3 | 4.4 | 3.6 | 0.83 |  | 65.2 | 15.5 | 0.24 |  | 37.7 | | 15.7 | 0.42 |
| Case 4 | 462.1 | 403.2 | 0.87 |  | 398.2 | 266.4 | 0.67 |  | 150.2 | | 34.9 | 0.23 |
| Case 5 | 929.9 | 898.8 | 0.97 |  | 772.1 | 551.7 | 0.71 |  | 229.85 | | 188.4 | 0.41 |
| Case 6 | 6289.3 | 3947.3 | 0.63 |  | 2548.2 | 1314.4 | 0.52 |  | 431.1 | | 105.0 | 0.49 |
| Case 7 | 1463.6 | 1556.7 | 1.06 |  | 1377.3 | 1428.1 | 1.04 |  | 99.0 | | 39.0 | 0.39 |
| Case 8 | 65.4 | 45.4 | 0.69 |  | 118.6 | 42.1 | 0.36 |  | 60.0 | | 16.0 | 0.27 |
| Case 9 | 2514.9 | 1791.9 | 0.71 |  | 2067.9 | 1352.7 | 0.65 |  | 150.0 | | 59.0 | 0.39 |
| Mean±SD | 1357.4±1456.9 | 1009.3±1210.9 | 0.82±0.1 |  | 892.5±863.7 | 593.2±571.1 | 0.58±0.2 |  | 181.4±116.2 | | 90.4±72.2 | 0.46±0.2 |

SD, standard deviation
