# Supplementary material for: Touch imprint cytology with massively parallel sequencing (TIC‐seq): a simple and rapid method to snapshot genetic alterations in tumors
Source: Cancer Med. 2016 Oct 24;5(12):3426–36. doi: 10.1002/cam4.950 (PMC5224853; doi:10.1002/cam4.950)
Supplement: Supplementary file 6 — Table S4. Comparison of EGFR mutations detected by targeted sequencing and PCR‐Invader method. [file CAM4-5-3426-s006.docx]

**Supplemental Table 4. Comparison of *EGFR* mutations detected by targeted sequencing and PCR-Invader method.**

|  |  | **Targeted seq** | |  | **PCR-Invader** |
| --- | --- | --- | --- | --- | --- |
| **ID** | **Mutation** | **TIC** | **FFPE** |  | **FFPE** |
| Case 1 | E746_A750del | + | + |  | + |
| Case 3 | ND | ND | ND |  | ND |
| Case 4 | ND | ND | ND |  | ND |
| Case 5 | G719C | + | + |  | + |
|  | S768I | + | + |  | + |
| Case 7 | A750_I759del insGG | + | + |  | + |
| Case 9 | L858R | + | + |  | + |

ND, not detected.
